# Building organisations, setting minds: exploring how boards of Dutch medical specialist companies address physicians’ professional performance

**DOI:** 10.1186/s12913-022-07512-6

**Published:** 2022-02-05

**Authors:** Maarten P. M. Debets, Milou E. W. M. Silkens, Karen C. J. Kruijthof, Kiki M. J. M. H. Lombarts

**Affiliations:** 1 Research Group Professional Performance and Compassionate Care, Department of Medical Psychology, Amsterdam University Medical Centres, University of Amsterdam, Amsterdam, the Netherlands; 2 Amsterdam Public Health Research Institute, Amsterdam, the Netherlands; 3 Research Department of Medical Education, University College London, London, UK; 4 Amsterdam University Medical Centers, Vrije Universiteit Amsterdam, University of Amsterdam, Amsterdam, Netherlands

**Keywords:** Physicians, Professional performance, Leadership, Healthcare governance and management, Quality and safety

## Abstract

**Background:**

Governments worldwide are reforming healthcare systems to achieve high quality and safe patient care while maintaining costs. Self-employed physicians reorganise into novel organisations to meet reconfiguration demands, impacting their work environment and practice. This study explores what strategies these novel organisations use to address physicians’ professional performance and what they encounter when executing these strategies to achieve high quality and safe care.

**Methods:**

This constructivist exploratory qualitative study used focus groups to answer our research question. Between October 2018 and May 2019, we performed eight focus group sessions with purposively sampled Medical Specialist Companies (MSCs), which are novel physician-led organisations in the Netherlands. In each session, board members of an MSC participated (*n* = 33).

**Results:**

MSCs used five strategies to address physicians’ professional performance: 1) actively managing and monitoring performance, 2) building a collective mindset, 3) professionalising selection and onboarding, 4) improving occupational well-being, and 5) harmonising working procedures. The MSC’s unique context determined which strategies and quality and safety topics deserved the most attention. Physicians’ support, trusting relationships with hospital administrators, and the MSC’s organisational maturity seem critical to the quality of the strategies’ execution.

**Conclusions:**

The five strategies have clear links to physicians’ professional performance and quality and safety. Insight into whether an MSC’s strategies together reflect medical professional or organisational values seems crucial to engage physicians and collaboratively achieve high quality and safe care.

**Supplementary Information:**

The online version contains supplementary material available at 10.1186/s12913-022-07512-6.

## Background

Governments in high-income countries are reforming healthcare systems to achieve high quality and safe care while maintaining costs [1, 2]. To meet reconfigurations demands, self-employed physicians reorganise themselves into new and often larger organisations, impacting their work environment and practice [3–7]. Self-employed physicians may find that by reorganising, they gain control over their work, allocate resources more efficiently [5], or create a strong collective that may offer more power to negotiate with hospitals [8, 9]. An example of such a novel organisation of self-employed physicians is the Medical Specialist Company (MSC) in the Netherlands. Self-employed physicians merged their specialty group partnerships into MSCs after the Dutch government introduced the Act on Integrated Funding in 2015 to optimise healthcare cost-efficiency (see setting). A typical MSC, of which there are around 70 in the Netherlands [10], comprises multiple mono-disciplinary specialty groups, has an exclusive relationship with one hospital, and is governed by a board of peers.

However, the success of MSCs and similar organisations is currently unknown and may depend on physicians’ involvement and organisational strategies enabling them to live up to their professional responsibilities [4, 11, 12]. The organisation might not function well if physicians are unwilling to take the lead or remediate peers [12, 13]. Conversely, physicians’ may be less likely to fulfil leadership positions and disclose performance information when the organisation fails to recognise the value of leadership and implement adequate performance monitoring systems, possibly harming the quality of patient care [4, 11, 14]. Moreover, physicians are primarily trained in medicine and may lack essential leadership skills and organisational knowledge needed for excellent professional performance in these new settings [15–18].

Therefore, it is problematic that we have limited insight into how novel physician-led organisations like MSCs achieve high quality and safe care [3, 5, 19]. More particularly, knowledge about effective strategies to address physicians’ professional performance is lacking [4, 11, 20]. Hence, this study will answer the following research question: What strategies do MSCs use to address physicians’ professional performance, and what do they encounter when executing these strategies to achieve high quality and safe care? Such information is especially relevant to boards of MSCs and similar organisations to guide their organisational development and improve their leadership and strategies to address physicians’ professional performance.

## Methods

### Setting

On January first 2015, the Dutch government reformed the payment model of medical specialist care. Before this time, self-employed medical specialists and hospitals billed their services separately, which was deemed more complex, difficult to regulate and costly [21]. The reform established integrated funding to align hospitals’ and medical specialists’ interests and improve healthcare quality and cost-efficiency. However, it also meant self-employed medical specialists lost their autonomous billing rights, a condition for the Dutch Tax Authority to qualify as an entrepreneur (i.e. self-employed) [22]. Without entrepreneurial status, they would lose their felt professional independence and eligibility for related tax benefits. Therefore, integrated funding drove most self-employed medical specialists to organise themselves in MSCs to maintain their entrepreneurial status and independence. Another effect was that MSCs and hospitals were required to make formal agreements about the quality and safety of patient care. Initially, MSCs devoted most of their efforts to the legal aspects of reorganising into MSCs [21]. However, more recent policy evaluations indicate that MScs increasingly focus on quality and safety [22].

While various MSC types and organisational formats exist, such as regional MSCs or MSCs including only one professional discipline, this study focuses on typical MSCs. Each MSC consists of multiple medical specialties and represents its members with a chosen board of peers that collaborates and negotiates with the hospital’s administration about various aspects of medical specialist care. The collaboration and specific negotiated agreements between MSCs and the hospital are contractually regulated. While hospital administration is ultimately accountable for all aspects of quality and safety by law, the MSC board is responsible for realising the goals stated in the contract.

### Ethics and consent to participate

All procedures were in accordance with the Helsinki Declaration. The institutional ethical review board of the Amsterdam UMC provided a waiver declaring the Medical Research Involving Human Subjects Act (WMO) did not apply to the current study (ref. W18_082#18.106). All participants provided written informed consent to participate.

### Study design

This constructivist exploratory qualitative study used focus groups [23] to answer our research question: What strategies do MSCs use to address physicians’ professional performance, and what do they encounter when executing these strategies to achieve high quality and safe care? By constructivist, we mean that knowledge is constructed from the experience and interaction between participants and the researcher. Focus groups are well suited within a constructivist paradigm and are particularly appropriate for exploratory research [23, 24].

### Research team

The team consisted of researchers from various disciplines; all are familiar with physicians’ professional performance research: a social scientist with a background in Strategic Human Resource Management (MD); a health scientist and research fellow in medical education within the NHS; a healthcare services researcher well-established in healthcare policy and management (KL) and a hospital administrator (KK) with a background in medicine and public administration. KK has a long-standing experience as hospital administrator of a teaching hospital where she witnessed the introduction of an MSC and collaborated with the MSC for several years.

In addition to the research team, we consulted two experts to further inform the development of the discussion guide and data collection: 1) a non-executive board member of a large teaching hospital collaborating with an MSC and 2) a policy consultant from the Dutch Association of Medical Specialists charged with assisting MSC boards.

### Sampling and inviting participants

We purposively selected MSCs varying in the number of adjoined medical specialists, hospital size, and geographic region to ensure maximum variation in our sample. We selected MSCs iteratively, meaning that each focus group session informed the next inclusion. KL and KK used available contact credentials to invite MSC chairs by email to participate in a focus group session, or MD approached the MSC board’s secretary to acquire contact credentials. Entire MSC boards were invited. Upon interest, MD provided a participation information letter by email.

### Participants

Half of the approached MSC boards agreed to participate in a focus group discussion (*n* = 8); others indicated lacking time or having other priorities. MSCs’ board sizes varied between three and seven members, and a professional staff supported all but one MSC. In four of the focus group sessions, the entire MSC board participated. Other sessions included all but one board member (*n* = 3) or half of the board and two supporting staff members (*n* = 1), resulting in 33 participants (28 physician board members, 5 non-physician board members and support staff). Table 1 provides a brief description contextualising the participating MSCs.

**Table 1:** Descriptions of participating MSCs

	≈ Number of specialists	Board size*	Hospital size**	Hospital recently merged***	Hospital location
MSC1	250	Large	Medium	Yes	Peripheral
MSC2	250	Small	Large	Yes	Peripheral
MSC3	150	Small	Medium	No	Urban
MSC4	50	Small	Small	No	Peripheral
MSC5	350	Large	Large	Yes	Peripheral
MSC6	250	Medium	Medium	No	Urban
MSC7	200	Medium	Large	Yes	Urban
MSC8	200	Large	Large	Yes	Urban

^*^Board size: small (< 4), medium (4), large (> 4)

^**^Hospital size in number of employees and yearly patient visits: small (< 2000 & < 250.000), medium (2000-4000 & 250.000-500.000), large (> 4000 & > 500.000)

^***^Recently merged meant merged within the last 5 years at the time of data collection

### Data collection & discussion guide

From October 2018 until June 2019, we conducted all focus group sessions in the hospitals of participating MSCs. To answer our research question, we developed a discussion guide consisting of three main parts (Additional file 1): 1) MSCs’ most critical quality and safety issues, 2) strategies to address physicians’ professional performance, and 3) MSC boards’ relationships with physician members and the hospital’s administration. Part one was introductory, part two addressed the main research question, and part three provided contextualisation. Policy evaluations and expert consultations pointed to the importance of part three to effectively address physicians’ professional performance.

A moderator (KL, MS) led the 60-75-min sessions; an observer (MD) took notes about verbal and nonverbal communication. The observer and moderator reviewed and discussed each focus group session directly following the meeting. All sessions were audio-recorded and transcribed. No financial compensation for participation was provided.

### Data analysis

Two researchers (MD, MS) independently read and open coded the first transcript and compared and discussed labelling and interpretations of codes until consensus. Next, MD coded the first three transcripts and grouped similar open codes to establish categories. Subsequently, the research team discussed initial categorisation to identify the most significant themes (axial coding). The remaining transcripts were double coded by MS in sections relevant to the research question. Lastly, the research team used a selective coding process to establish the interconnectedness between themes, i.e. noting all main themes and discussing mutual relationships.

The research team discussed the outcomes of all coding stages extensively. After focus group session eight, the categories seemed to manage the data without further modifications, indicating theoretical sufficiency [25]. MAXQDA was used to support all analyses.

## Results

We found five strategies that MSCs used to address physicians’ professional performance to achieve high quality and safe care: 1) actively monitoring and managing performance, 2) building a collective mindset, 3) professionalising selection and onboarding, 4) improving occupational well-being, and 5) harmonising working procedures. MSC boards seemed to agree that excellent performance included being a medical expert and team player, actively participating in the MSC, and understanding organisational processes in the MSC and hospital.

Table 2 provides insight into how MSCs address physicians’ professional performance (i.e. an overview of strategies, efforts, instruments, or tools used), whereas the elaboration on each strategy below describes why they are needed and *what* happens when applying them.

**Table 2:** Overview of reported specific initiatives within the five identified strategies used by MSC’s to address physicians’ professional performance

1. Monitoring and managing performance	2. Building a collective mindset	3. Prof. selection & onboarding	4. Improving occupational well-being	5. Harmonising working procedures
Individual and group performance appraisals	Continually emphasising shared goals	Introduction programs	Offering social support	Harmonising medical protocols and guidelines
Soft signal systems	Appointing specialty group representatives with a collective mindset	New recruitment and selection methods (e.g. 360 degrees feedback)	Referring to professional help	Harmonising well-being practices and policies
Specialty group visits	Company drinks and party’s	Monitoring the need for vacancies within specialty groups	Initiating occupational well-being programs	Offering insight in MSCs policies and practices relevant to all members
Assisting specialty group representatives	Introducing a company website and newsletter	Buddy systems for new members	Career path polices	Codes of conduct for all members
Role modelling	Changing the physical setting of meetings to involve members	Checking the qualifications of new members rigorously	Flexible working (less night shifts, hours)	Offering to facilitate insurance for all members
Speaking-up to members about their performance				
Financial compensation or sanction				
Mediation trajectories				
Remediation trajectories				

Note: The presented initiatives can serve multiple goals and strategies but are depicted under the most relevant strategy

### 1. Actively monitoring and managing performance

MSC boards monitored and managed various aspects of physicians’ professional performance to keep track of quality and safety. The most substantial aspect was specialty group’s patient care volume and related quality indicators. Less frequently monitored aspects were compliance to hand hygiene and physicians’ professional learning goals and well-being. The topics that deserved priority depended on MSCs’ local context, e.g. outcomes of quality and safety audits*.* However, MSCs also reported a series of challenges when executing this strategy: depending on physicians’ willingness to share performance information, having insufficient insight into ‘softer’ performance aspects, and managing unprofessionalism.

MSC boards reported that physicians were not always willing to share performance information, especially when this involved reporting peers: “It is a bit of betrayal of your mate, and you don’t do that lightly (*MSC6, R2*)”*.* Another worry was that MSC boards might “reduce your autonomy (*MSC6, R1*)” and interfere with practice. Some specialty groups would rather cover-up performance issues and let them evolve into more significant problems before consulting the MSC board. “Well, it is very much covering-up (…) we’ve seen it coming for years that it is just not good [enough]. And then they get a bad audit, and suddenly they do come to you (*MSC6, R3*)”*.*

Related to this, MSC boards reported to mainly have sight on extreme incidents and missed “a clear overview of all specialty groups, if there are indeed, those soft signals (MSC2, R1)”. Soft signals referred to early indications of performance issues, e.g. a colleague starting to communicate unfriendly to other healthcare professionals. Therefore, MSCs reported initiating specialty group visits to exchange performance information with physicians and so-called soft signal systems “to prevent that a remediation trajectory needs to follow (MSC1, R4)”. While soft signal systems allowed hospital actors to report concerns about physicians’ professional performance, MSC boards indicated implementation and widespread use would take time.

MSC boards considered managing physicians’ unprofessional behaviours particularly challenging as these behaviours were often ambiguous, physicians thought differently about ‘unprofessionalism’, and related protocols and procedures were vague. MSC boards said: “The consequences for certain behaviours or the absence of good behaviour in the field of quality and safety are really lacking (MSB2, R2)”. They reported that this related to the MSC’s non-hierarchical structure: “You have no boss above you and you have a lot of freedom. But if there is a hitch somewhere (…) you have no instruments to do anything (MSC6, R3)”. More clear cut procedures were available for managing ‘dysfunctioning’ medical specialists, which was a joint task of the MSC board and hospital administrators requiring mutual trust. MSC boards reported that a lack of trust prevented effective monitoring and managing: “In theory, this could mean that I delay reporting a dysfunctioning medical specialist, that is the effect if you do not have mutual trust (MSC3, R2)”.

When executing this strategy MSC boards mainly focused on underperforming physicians and specialty groups: “Before you know it, you are only looking at problem situations, and you are not hearing the specialty groups that are doing quite well (MSC7, R2)”. MSC boards occasionally mentioned sharing best practices or financially rewarding desired behaviours. Trustworthy relationships with the hospital’s administration, the boards’ leadership competencies (e.g. conflict management skills), and the implementation phase of performance monitoring systems appeared critical for the effectiveness of this and the other strategies reported below.

### 2. Building a collective mindset

MSC boards worked towards building a collective mindset, which meant physicians were encouraged to rise above their own speciality group’s perspective and adopt a hospital-wide focus. A collective mindset was considered crucial to create buy-in from physicians, facilitate best practice sharing, openness to new ways of working, and inter-disciplinary collaboration. For this strategy, specialty group representatives were vital because they acted as a linking pin between the MSC board and the work floor and “know what is going on, behind them is a group [of MSC members] that probably doesn’t know what’s going on, they absolutely rely on what their specialty group representative tells them to vote (MSC3, R2)”. Participants reported appointing specialty group representatives with a positive attitude towards the MSC to foster a collective mindset (Table 1).

However, MSC boards described achieving this hospital-wide thinking as “our biggest challenge (MSC1, R4)” and that “it is not so much about the hospital, and you notice that in all specialty groups (MSC6, R1).” They wanted to facilitate “the translation to a really actively participating medical specialist in this company, that this is also their company, their nest, and that you have to keep it very good. Some think that is logical, but a large part sees it different (MSC6, R3)”. According to participants, building a collective mindset was challenging due to physicians’ perceptions and fears of losing autonomy, influence or resources since the foundation of the MSC. Some specialty groups would say: “Previously we could arrange things for ourselves much better than the MSC can arrange it for us (MSC7, R1)”, while MSC boards aimed “to do the best for the corporation (…) So they must give up some [profit] for the others who had arranged it less well (MSC7, R3)”. Also, groups were no longer allowed to make individual arrangements with the hospital administration: “All arrangements that are made with specialty groups run through the MSC (…) especially specialty groups that frequently dealt directly with the hospital administration see it as an obstacle (MSC7, R3)”. Trustworthy relationships with the hospital’s administration prevented that specialty groups were able to bypass the MSC board.

MSC boards sought adequate tools and leadership styles to build a collective mindset. They learned by trial and error, and some experienced the benefits of more actively involving physicians in decision making. This strategy assisted in obtaining physicians’ support, which MSC boards indicated as crucial in a company among equals in which members vote for MSC proposals with potentially adverse consequences for themselves or their specialty group. Therefore, physicians’ support was a critical factor for executing all strategies.

### 3. Professionalising selection and onboarding

MSCs improved procedures of selecting new physicians and supported integration in the MSC and hospital. They used this strategy to select qualified physicians and obtain enhanced insight into their ambitions to participate in quality and safety committees or leadership positions. MSCs also employed this strategy to foster physicians’ organisational awareness. They said to find it important “that people are more aware of the organisation in which we work (MSC3, R2)” and have an idea of “what a manager actually does and why he is not only a burden (MSC5, R1)”.

MSC boards described coordinating open vacancies to more professionally recruit and build the medical staff: “Now an extra pair of eyes is watching; is that vacancy really necessary? (MSC1, R1)”. They also discussed implementing more rigorous selection procedures, such as “a built-in reference check (…) the hospital and adjacent specialties provide 360-degree feedback and that determines whether someone should be welcomed definitely (FG3, R3)”. Regarding integration, participants mentioned offering leadership and management training, and MSC6 supported integration using a buddy system, facilitating social support and opportunities to build a professional network in the hospital.

Still, sometimes it was challenging to achieve the expected outcomes. For example, one MSC described not reaping the benefits of sending physicians to a leadership program because they “chose people who were too young (MSC3, R2)”, meaning they already had too much on their plate (e.g. children, high workload). Furthermore, some MSCs struggled to attract and attain qualified medical specialists “because we are located in a region that is less attractive to the average doctor (MSC1, R1)”, which for them enhanced this strategy’s importance.

### 4. Improving occupational well-being

MSC boards said to work on improving physicians’ occupational well-being, encouraged by well-being featured in the media and the realisation that “apart from the personal misery, it also has consequences for quality and safety (MSC5, R1)”. Most MSC boards mentioned offering informal and professional help to peers on sick leave or those at risk of dropping out. However, they also expressed their ambitions to be more proactive, for example, by aligning physicians’ professional preferences and working conditions: “How can we ensure that everyone gets their right place, (…) you enter a different life phase, in which you want to have children or want to do other things, and how do you create room for that, we are working that out in an HR working group (MSC2, R1)”.

Nonetheless, such initiatives were infant, and MSC boards described several complexities in addressing occupational well-being, such as “how generic can you make it [flexible night shift policies], because the conditions differ per specialty group (MSC5, R1)” and “if he starts doing fewer night shifts, that means another colleague will be charged more (MSC4, R2)”. Also, participants contemplated how to deal with (younger generation) physicians refusing to perform MSC tasks to achieve a better work-life balance. Lastly, some MSC boards said “I notice that we are actually increasingly taking the role of employer (MSC5, R3)” and questioned their role and responsibilities for addressing well-being in an organisation of self-employed physicians.

### 5. Harmonising working procedures

MSC boards reported harmonising work procedures that differed between specialty groups to benefit patient care. This was especially true for MSCs of merged hospitals, for example, when harmonising work procedures of two gastroenterologist groups: “Then you have the rules of two professional associations in your specialty group (…) they have different opinions on sub-topics, and what opinion will you follow? You have to be audited by two associations as one specialty group (…) and we saw all kinds of problems occur that we did not find acceptable (MSC7, R3)”. Therefore, MSC7 financially supported the training of physicians enabling them to work according to one professional guideline. MSC boards indicated that executing this strategy could be challenging as some specialty groups would like to stick to their ways of working.

Furthermore, this strategy included providing “more clarity and information, that we can offer a solution together, that it is not a problem of the individual alone or the specialty group. (MSC2, R1)”. MSC boards indicated that physicians were often unaware of MSC-wide policies, regulations and resources.

#### Conceptual model

**Fig. 1:**
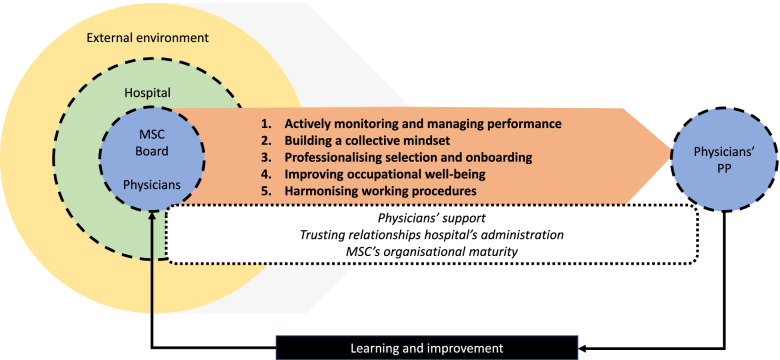
Conceptual model

Figure 1 summarises our findings and depicts the five strategies MSCs used to address physicians’ professional performance to achieve high quality and safe care. Furthermore, it shows three critical factors to the overall functioning of the MSC and the quality of the strategies’ execution: physicians’ support, trusting relationships with hospital administrators, and the MSC’s organisational maturity. Without physicians’ support, the board lacked administrative power to govern the organisation. Trustworthy relationships with the hospital administration were crucial to manage dysfunctioning physicians and negotiate preferred working circumstances overall. Organisational maturity refers to aspects such as MSC boards’ leadership competencies and the implementation phase of performance monitoring systems. The model shows that MSC’s unique context influenced which strategies and quality and safety topics deserved the most attention. The feedback loop illustrates that MSC boards may learn from addressing physicians’ professional performance and improve their strategies accordingly.

## Discussion

This study explored what strategies Dutch Medical Specialist Companies (MSCs) – novel physician-led organisations – use to address physicians’ professional performance and what they encounter when applying these strategies to achieve high quality and safe care. The empirical analysis revealed five strategies: 1) actively monitoring and managing performance, 2) building a collective mindset, 3) professionalising selection and onboarding, 4) improving occupational well-being, and 5) harmonising working procedures. We first discuss how each strategy may relate to the quality and safety of patient care. Then, we adopt a strategy-overarching perspective discussing how MSCs addressed physicians’ professional performance concerning their main challenge.

Without monitoring and managing performance, it is impossible to know whether physicians’ performance is consistent with quality and safety standards. However, our results indicate that effective monitoring was hampered by physicians’ reluctance to share information, which stemmed from protecting peers or their autonomy. Prior studies reported similar reasons for physicians not reporting incidents, such as a rejection of bureaucracy and a culture of protectionism [26, 27]. Also, MSCs seemed predominantly aware of extreme incidents, which provides an incomplete picture of quality and safety [28, 29]. Literature suggests that soft signals, previously described as observable deviations from a colleague’s regular professional performance, are vital for comprehensively assessing patient safety risks [29]. Although MSCs described initiating such soft signal systems, they rarely discussed creating a blame-free culture essential for hospital actors to report soft signals [28, 29]. Concerning managing, addressing unprofessionalism seemed particularly challenging due to unclear protocols, varying views on professionalism, and a lack of tools. However, not acting on unprofessionalism may undermine a safety culture and increase risks of medical errors and surgical complications [30].

Building a collective mindset can improve quality and safety by aligning MSC’s vision and strategies with specialty groups’ practice [31, 32]. This alignment was crucial for novel hospital-wide committees to contribute to continuous quality improvement in postgraduate medical education [32]. A shared vision can also guide goal-setting, which motivates and contributes to performance [33]. In the context of MSCs, participatory goals setting might contribute to ownership and active involvement [33], hence the quality and safety provided in hospitals [12].

The strategy professionalising selection and onboarding might foresee in recruiting physicians with leadership aspirations who underscore the vision and values of the MSC [34]. A stronger person-organisation fit correlates with reduced turnover intentions, increased affective commitment, job satisfaction and organisational citizenship behaviours [35, 36]. MSC boards also described using integration to get more insight into physicians’ professional preferences and leadership ambitions, which can help create a leadership pipeline needed to cope with future challenges [37]. Moreover, such information can be used to design physicians’ ‘jobs’ in personally meaningful ways [38, 39], potentially improving their well-being and performance [40, 41].

That improving occupational well-being relates to the quality and safety of patient care is well underpinned by research, leading researchers to describe physicians’ well-being as ‘a quality indicator’ [42]. Ample evidence shows that physicians’ occupational well-being contributes to better patient satisfaction and interpersonal aspects of care [43]. There also seems to be a link between physicians’ occupational well-being and patient outcomes, but the evidence rests mainly on self-reported data [44]. Studies indicate that sustainably improving physicians’ occupational well-being requires proactive efforts from physicians and their organisations [45, 46].

Unharmonised working procedures endanger patient care as complicated, inaccurate, unrealistic, absent or poorly presented protocols cause adverse events in hospitals [47].

From a strategy-overarching perspective, MSC boards identified creating an actively involved cadre of physicians with a hospital focus as their primary challenge. Our results indicate that this is a cultural challenge originating in physicians’ perceptions of losing autonomy, influence or resources since the formation of the MSC. Physicians may perceive the MSC board and its strategies, despite the board’s collegial nature, as an intrusion of organisational logics into the medical domain. Organisational leadership generally endorses values like control, costs and efficiency, which physicians may see as detrimental to medical professional values, e.g. providing high quality and compassionate care [48–50]. Managing these conflicting values requires excellent leadership skills, and inexperienced leaders often feel more comfortable controlling physicians’ performance based on rationality than intervening on ambiguous cultural performance aspects [17, 51, 52].

Although MSC boards acknowledged the importance of intervening on culture, they regularly used descriptions that seem to reflect organisational logics and ambitions to control physicians’ professional performance, potentially reinforcing their main challenge. Control-based approaches to managing performance encompass compliance to rules, supervision and autocratic decision making [53, 54]. For example, MSC boards described focusing on poor performance and wanted instruments to punish unprofessionalism. Another example is that an MSC board initially did not consider physicians’ workload before ‘sending’ them to a leadership development program. While MSC boards seemed to have the best intentions, these examples may unintendedly convey to physicians ‘we do not trust you’ or ‘your time and well-being are less valuable than organisational priorities’ ([55], p.1558). Due to value dissonance, a control orientation may result in a loss of physicians’ support, impede organisations to learn from what is going well, and create a work environment in which burnout thrives [54, 56, 57]. Commitment-based approaches steer physicians’ performance by developing skills, motivation, sharing best practices, facilitation, and participatory leadership [53, 54], and align better with professional medical values [14, 49, 58, 59]. When MSC boards aim to commit physicians and ensure the employed strategies reflect their intentions, they might address physicians’ professional performance more effectively.

### Strengths and limitations

Strengths of this study are the inclusion of differently composed ‘typical’ MSCs throughout the Netherlands and the participation of entire MSC boards. These strengths contributed to in-depth insight into the perspectives of and interaction between board members within MSCs, and how different MSCs address physicians’ professional performance.

Including the perspective of MSC boards only can also be a limitation, as their opinions might not completely represent what is happening in practice, or at some points may be perceived differently by their physician members.

Lastly, the unique Dutch setting of this study might be seen as a limitation. The findings may not be transferable to other contexts one-on-one. However, this study provides more insight into the complexities of leading novel physician organisations and might inform strategies to address physicians’ performance to improve patient care.

### Implications for research and practice

Future research could focus on discrepancies between MSC physician members’ and MSC boards’ perspectives on managing professional performance. Understanding such discrepancies is essential for achieving high quality and safe care because physicians presumably act on their perceptions of initiatives rather than on how boards intended them [14, 55]. More specifically, studies could unravel under what circumstances physicians’ experience the board’s actions in line with medical professional or organisational logics. Lastly, quantitative research could establish the link between MSCs’ strategies and patient outcomes or experiences.

For practice, this study indicates that it might be beneficial for boards of novel physician organisations to actively address culture next to building organisational structures, systems and guidelines. Moreover, it seems necessary to evaluate whether the strategies used resonate with the MSC’s vision and intentions to address physicians’ performance. Leadership training programs could help physician board members to develop the needed skills. They could also offer leadership training to physicians, particularly specialty group representatives, to foster organisational awareness and involvement.

## Conclusions

This study explored how MSCs address physicians’ professional performance to achieve high quality and safe care and identified five strategies. The identified strategies have clear links with professional performance and quality and safety. Considering whether the strategies reflect medical professional or organisational values might help create a cadre of actively involved physicians with organisational awareness. Future research on MSC physicians’ perspectives is needed to obtain a more balanced understanding of MSCs’ practice.

## Supplementary Information

**Additional file 1.** Discussion guide.

## Data Availability

The datasets generated and analysed during the current study are not publicly available due to its qualitative nature and potential to compromise participants’ anonymity. Please contact the corresponding author [MD] for specific inquiries about the data substantiating the results of this study.

## Abbreviation

MSC: Medical Specialist Company
