# Supplementary material for: Building organisations, setting minds: exploring how boards of Dutch medical specialist companies address physicians’ professional performance
Source: BMC Health Serv Res. 2022 Feb 5;22:155. doi: 10.1186/s12913-022-07512-6 (PMC8818234; doi:10.1186/s12913-022-07512-6)
Supplement: Supplementary file 1 — Additional file 1. Discussion guide. [file 12913_2022_7512_MOESM1_ESM.docx]

**Additional file 1: Discussion guide**

*Introduction*

- Please motivate your answers from the perspective of a MSC board member
- Please specifically keep the initiatives proposed by the MSC aimed at enhancing physician members’ professional performance in order to achieve high quality and safe care in mind throughout your participation.
- Could you please tell me your name and current position?

*Key questions*

Part 1

- What do you consider to be the three most important professional quality and safety topics during the past year?
  - Each board member writes down his or her most important themes. Next, the moderator facilitates plenary discussion using similarities and discrepancies between the topics noted by board members.

Part 2

- How do you ensure that physician members can perform optimally within the MSC?
- How do you deal with [case presented] or [physician members’ occupational well-being]?
  - The moderator hands over case descriptions of fictional MSC physician members to the participants (see the case descriptions below).

Part 3

- How would you position the MSC board in relation to physician members’ and the hospital’s administration?
  - The moderator hands over stickers and a sheet of blanc paper (see example below) to each board member to indicate how they perceive the relationship – focusing on position and distance-with physician members and hospital administrators. Each board members explains his or her sheet of paper, opening up the conversation.

Wrap-up

- Are there topics that have not been discussed but are essential for us to know in the light of this study?

*Case descriptions*

| Edwin, 58 years old, has been working as a neurologist for 25 years. Lately, he has been going to work with less pleasure and enthusiasm. He increasingly experiences the evening and night shifts as a (physical) burden and notices that his capacity to recover is diminishing. He has difficulty adapting to the many workplace changes, such as remote consultations, working with new administrative systems, and the many quality requirements imposed on him and his colleagues. Due to (marital) problems at home, he hardly gets rest there.  Yvonne is 37 years old and has been working as a surgeon for five years now. She does her job well, is ambitious, and fits well within the team. Yvonne notices that after five years, she needs more flexibility in her work - she has two young children at home. She is broadly interested and would like to do doctoral research. She wonders what the possibilities are within her current position. Yvonne is satisfied with her job, but regularly receives offers from other hospitals. |
| --- |

*Stickers on blanc paper*

| 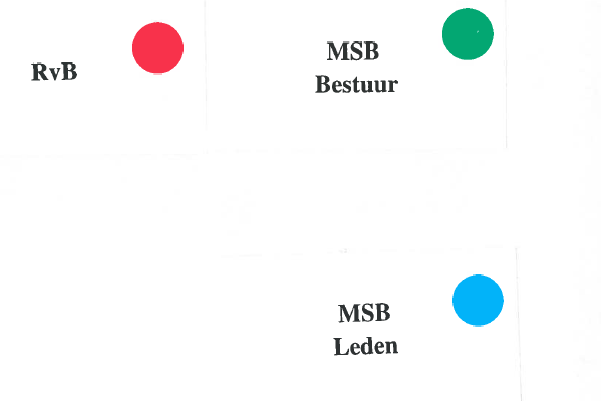 |
| --- |
| RvB = hospital administration  MSB Bestuur = MSC board  MSB Leden = MSC physician members |
